# Some Open Mathematical Problems on Fullerenes

**DOI:** 10.1021/acs.jcim.4c01997

**Published:** 2025-03-10

**Authors:** Artur Bille, Victor Buchstaber, Evgeny Spodarev

**Affiliations:** † Ulm University, 89069 Ulm, Germany; ‡ Steklov Mathematical Institute RAN, 119991 Moscow, Russia

## Abstract

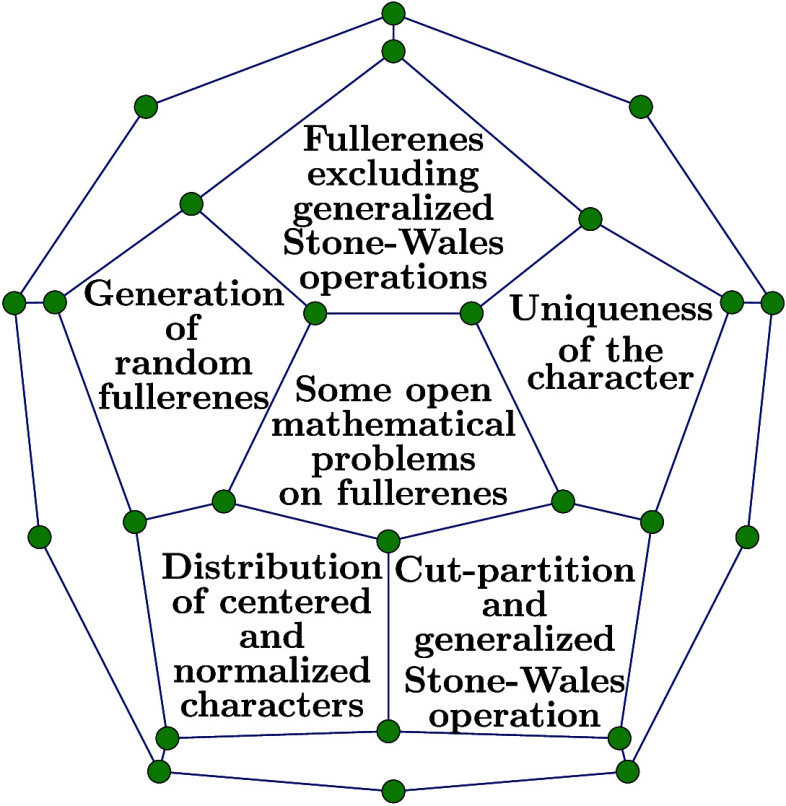

Fullerenes are hollow carbon molecules where each atom is connected to exactly three other atoms, arranged in pentagonal and hexagonal rings. Mathematically, they can be combinatorially modeled as planar, 3-regular graphs with facets composed only of pentagons and hexagons. In this work, we outline a few of the many open questions about fullerenes, beginning with the problem of generating fullerenes randomly. We then introduce an infinite family of fullerenes on which the generalized Stone–Wales operation is inapplicable. Furthermore, we present numerical insights into a graph invariant, called the *character* of a fullerene, derived from its adjacency and degree matrices. As supported by numerical results, this descriptor may lead to a new method for linear enumeration of all fullerenes.

## 1 Introduction

Fullerenes, spherical molecular structures composed of pentagonal and hexagonal rings of carbon atoms, have long captivated researchers across various disciplines. Shortly after their discovery, the enumeration and counting of fullerenes became a subject of high interest among chemists, physicists, computer scientists and mathematicians which has led to a substantial body of research. Despite decades of study, several fundamental questions regarding fullerenes, particularly from a mathematical perspective, remain unresolved. This paper aims to address some of these open problems by presenting hypotheses based on numerical evidence, which can be replicated using the algorithms provided in ref (8). The insights and algorithms discussed here may serve as the groundwork for further research in the mathematical analysis of fullerenes. A notable example of a proof assisted by computer computations of a conjecture, long unresolved until recent computational advances, is the *Barnette-Goodey conjecture*. Originally stated in the 1960s, this conjecture asserts that all fullerene graphs are Hamiltonian. In other words, every fullerene graph contains a closed path that passes through every vertex of the graph once without repeating any vertices, except for the starting and ending vertex. In 2017, Kardoš, who had previously proven the sharpest lower bound for the number of vertices in the longest path on a fullerene graph,^24^ provided a valid computer-assisted proof of the Barnette-Goodey conjecture in ref (33).

In 1985, Curl, Kroto, and Smalley demonstrated the existence of a novel carbon allotrope, which they named *fullerene*. For this discovery, they were awarded the Nobel Prize in Chemistry in 1996. A year before that, Fowler and Manolopoulos^27^ offered a comprehensive overview of the theory and mathematics behind fullerenes. This influential work outlined open fundamental mathematical questions related to fullerenes as well as their physical and chemical properties. Additionally, they introduced the important *face spiral conjecture*, which offered the first general framework for generating and enumerating (mathematical) fullerenes.

Graph theory has proven to be an indispensable tool for a detailed analysis of fullerene structures. In 2015, Schwerdtfeger et al. published a very comprehensive review on recent topological and graph-theoretical developments, along with open problems in this area.^44^ Many of the problems highlighted in their work remain unsolved, and some are revisited in this paper from a slightly different perspective. Their review provides an extensive overview of fullerenes, emphasizing mathematical aspects while also addressing physical and chemical considerations, such as the thermodynamic stability and gas-phase formation of these molecules. Additionally, Schwerdtfeger et al. provide a substantial list of references, which we highly recommend as a starting point for readers who want to explore specific topics in greater depth. Since then, significant progress has been made and the list of references can be updated with many more publications. For example, in 2016, Andova et al.^2^ provided an overview of graph invariants and their potential correlation with the stability of the fullerene molecules. A subsequent study by Sure et al.,^46^ published just one year later, expanded this analysis from a chemical and physical perspective, employing high-accuracy quantum chemistry methods to compute the relative energies of all *C*_60_ isomers. The study identified connections between these results and various topological indices and geometrical measures, ultimately proposing a list of *good stability criteria*. In ref (11), the *Newton polynomials* of (subgraphs of) dual fullerene graphs were introduced as another good stability criterion, based on the spectra of the adjacency matrix *A* of these graph representations.

Since Newton polynomials can be interpreted as the counts of closed paths on the fullerene graphs, they are closely linked to many other chemical and physical properties of the corresponding molecules, as shown by Tsuji et al.^48^

In the present paper, we extend these results by incorporating a diagonal matrix *D*, which contains the degree of each vertex of the considered graph along its diagonal and which we refer to as the *degree matrix*. We examine the linear combinations, α*A* + β*D*, and introduce a novel graph invariant termed the character of fullerenes. This new invariant has the potential to address some of the open problems outlined here and in ref (44).

From a chemical and material science point of view, Xu et al. recently published a comprehensive review article on the latest developments in fullerene chemistry.^19^ In addition to discussing conventional fullerenes as defined in this paper, Xu et al. also explore a wide range of fullerene-like molecular structures, presenting new opportunities for mathematical investigation in the future. We highly recommend this article, particularly for its exceptionally extensive list of references. Another noteworthy overview article by Hirsch provides a broad summary of fullerenes, nanotubes, and graphene, with a focus on their electronic, thermal, and mechanical properties.^32^

The mathematical methods presented in this paper, such as the polyhedral Stone–Wales operation and the (α, β)-character, can be adapted to other molecular classes beyond fullerenes. Fullerenes should not be viewed merely as carbon-based molecules but rather through the lens of their underlying structural properties. Notable fullerene structures include *Goldberg polyhedra*,^28^ the hexagonal lattice,^9^ and nanotubes,^10,51^ each characterized by unique topological features that exemplify key fullerene forms. These structures find applications beyond chemistry, physics, and mathematics. For instance, the hexagonal lattice is employed in modeling complex cellular mobile communication networks, where its properties help in network optimization.^34^ In biology, Goldberg polyhedra have been used to model the shapes of small viral capsids, as noted in ref (35) and its references.

This paper is structured as follows. First, we revisit and extend the concept of the face spiral in the context of methods for constructing fullerenes at random, drawing on the approaches discussed in ref (41), and review additional algorithms for constructing fullerene isomers, including the *Stone–Wales operation* and two of its generalizations, one of which will be examined in more detail.

Subsequently, we present novel findings on the eigenvalues of linear combinations of adjacency and degree matrices representing (sub)graphs of fullerenes. Using these eigenvalues, we introduce a graph invariant, which allows for a linear ordering of fullerenes within *C*_*n*_ and across all feasible values of *n*. This ordering provides an alternative approach enumerating fullerenes, compared to the commonly accepted method based on the face-spiral hypothesis.

## 2 Preliminaries

We begin by recalling important definitions and notation used throughout this article.

A *fullerene* is a convex, 3-regular polytope consisting only of pentagonal and hexagonal facets. Let *n* denote the number of vertices in a fullerene. By Euler’s polyhedron formula and Eberhard’s theorem,^29^ every fullerene contains exactly 12 pentagonal facets, while the number of hexagons *n*/2–10 depends solely on *n*. Consequently, *n* must be an even integer for at least one fullerene with *n* vertices to exist. Notably, no fullerene with *n* = 22 vertices exists, as shown in ref (30). Thus, we define a positive even integer *n* ≥ 20, *n* ≠ 22, as *feasible*.

Let *C*_*n*_ denote the set of all fullerenes with exactly *n* vertices. This set can be partitioned into equivalence classes under graph isomorphisms, with each class referred to as a *C*_*n*_*-isomer*. Additionally, let  represent the union of all *C*_*ñ*_ with *ñ* ≤ *n*.

By Steinitz’s theorem,^29^ fullerenes can be associated with planar graphs that preserve their combinatorial structure and properties. A graph *G* is defined as a tuple *G* = (*V*(*G*), *E*(*G*)), where *V*(*G*) is the set of vertices, and *E*(*G*) the set of edges. Each edge is an unordered pair of vertices (*v*, *w*) with *v*, *w* ∈ *V*(*G*). A *facet* of the graph *G* is a connected region of the plane enclosed by a *closed path* of vertices (*v*_1_, ..., *v*_*l*_), *l* ≥ 2, where (*v*_1_, *v*_*l*_), (*v*_*j*_, *v*_*j*+1_) ∈ *E*(*G*) for *j* = 1, ..., *l* – 1. We define one facet as being *larger* than another if it is enclosed by more vertices. Additionally, a vertex *v* ∈ *V*(*G*) is called a *boundary vertex* of a facet *f* if *v* is part of the closed path enclosing *f*.

A common graph representation of polytopes, like fullerenes, is given by the *Schlegel diagram*, being a projection of a 3-dimensional polyhedron onto the plane. Although this projection is not bijective (meaning that the resulting embedding of the graph is not unique, where uniqueness or equality (≃) of graphs is meant up to an isomorphism), the combinatorial properties of the graph are invariant. Henceforth, we identify a *fullerene* with its corresponding planar graph representation.

Define iso(*n*)≔ |*C*_*n*_| and  to be the number of fullerenes in *C*_*n*_ and , respectively.

The exact values of iso(*n*) are known for *n* ≤ 400 by generating all possible isomers, cf. ref (20). In 2023, Engel et al. derived a formula for a function whose value is at most twice as high as *iso*(*n*) for any feasible *n*, providing a good approximation of the number of fullerenes, cf. ref (22).

An asymptotic behavior was derived by Rukhovich,^42^ building on general insights from Thurston^47^ and Engel et al.^23^
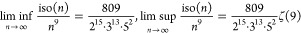
where ζ is the *Riemann zeta function*, with ζ(9) ≈ 1.00200839.

**Figure 1:**
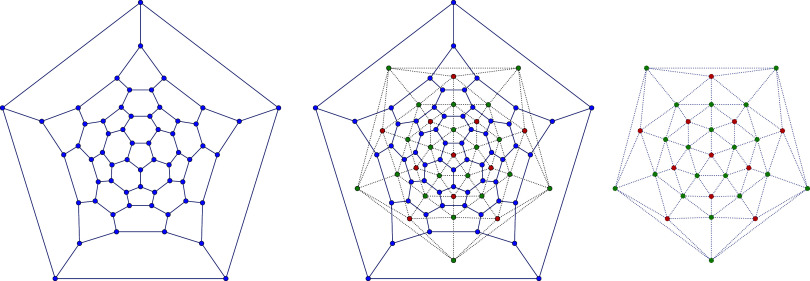
Buckminsterfullerene *C*_60,1812_ represented as a planar graph derived from the Schlegel projection (left) and its dual graph (right), with the relationship between the two illustrated in the center.

Since fullerenes are 3-connected, Whitney’s theorem (cf. ref (29)) ensures that their dual graph is unique. A *dual fullerene*, denoted by *T*_*n*_, can also be viewed as an element of the set of all convex triangulations of the sphere, as described in ref (22). In this article, we focus on dual fullerenes only, noting that all results can easily be translated in terms of the original fullerene structures. An example illustrating a planar graph obtained from the Schlegel projection, its dual, and the connection between them is presented in Figure 1. In the figures presented, vertices are color-coded as follows: blue for vertices of degree 3, green for vertices of degree 5, red for vertices of degree 6, and white for vertices with arbitrary degrees of 5 or 6.

The set of vertices *V*(*T*_*n*_) can be partitioned into two sets: one containing vertices of degree five and the other containing vertices of degree six. The subgraph of *T*_*n*_ induced by the vertices of degree five captures the connectivity between the pentagons, while the subgraph induced by vertices of degree six represents the connectivity between hexagons. We denote these graphs by *T*_*n*_^5^ and *T*_*n*_^6^, respectively.

Fullerenes with no edge connecting any pair of vertices in *T*_*n*_^5^ are said to satisfy the *Isolated Pentagon Rule* (IPR). These *IPR-isomers* are believed to be chemically more stable than non IPR-isomers, making them typically more deserving the detailed analysis.

The introduced graphs can be uniquely represented by their adjacency matrix *A*. Additionally, the degree matrix *D* (a diagonal matrix with degrees of all vertices on its diagonal), is often considered in graph theory, along with their linear combinations α*A* + β*D*, . For example, choosing α = −1 and β = 1 results in the well-known *Laplacian matrix* of a graph.

## 3 Random Fullerenes

The need to randomly select a fullerene uniformly distributed on *C*_*n*_ arises in many contexts, particularly in mathematical research. For example, a typical objective is to study the limiting behavior of the topological properties of sequences of fullerenes as the number of vertices *n* grows. The specific sequence of fullerenes chosen influences the limit, making the selection of its elements critical. To apply statistical methods, assumptions about the distribution of fullerene selection and the dependencies between chosen elements are typically required. This naturally leads to the following problem:
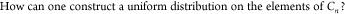


More precisely, for a fixed feasible *n*, we seek a random graph *X*_*n*_ ∈ {*C*_*n*,1_, ..., *C*_*n*,iso(*n*)_} such that
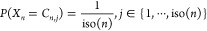
1without explicitly generating the set {*C*_*n*,1_, ..., *C*_*n*,iso(*n*)_} or knowing *iso*(*n*) in advance. These two restrictions are crucial, as will becomes evident in the next subsection. Once the behavior of *X*_*n*_ is understood, an efficient implementation of a function that generates samples of *X*_*n*_ is required.

An answer to the above-stated question may be related to the sizes of pentagonal clusters–i.e., the number of vertices in all connected components of *T*_*n*_^5^ – in a fullerene, which have been analyzed in ref (6). The critical partition of 12 into 12 ones corresponds to the aforementioned IPR fullerenes. Initially, in the study of fullerenes, only a few IPR fullerenes were known besides buckminsterfullerene *C*_60,1812_, leading to the assumption that IPR fullerenes were rare. However, Baši’c et al. demonstrated the existence of infinitely many IPR fullerenes (cf. ref (6)), and Rukhovich’s results imply that the fraction of IPR fullerenes in *C*_*n*_ approaches 1 as *n* → ∞ (cf. ref (42)). This finding implies that, for large *n*, the most frequently occurring partition in fullerenes is the one consisting of 12 ones. Furthermore, the authors of ref (6) established that 15 out of the 77 theoretically possible integer partitions of 12 are not realizable in fullerene graphs. Notably, all these unrealizable partitions involve a largest connected component in *T*_*n*_^5^ with at least six vertices. Despite these findings, several questions remain unanswered. For instance, which integer partition occurs second most frequently among all fullerenes? How does the distribution of isomers among integer partitions change when IPR fullerenes are excluded? Numerical experiments may provide valuable insights into the distribution of fullerene isomers *C*_*n*_ for a given *n* among all realizable partitions of 12. While analyzing the connection between the generation of random fullerenes and the partitioning of the 12 pentagons is an intriguing direction for future research, the approaches described below do not take the partition of pentagons into consideration.

As is immediately apparent, the construction of *X*_*n*_ requires an efficient algorithm for generating fullerene graphs. This algorithm must be capable of constructing every isomer, meaning it must produce different outputs. To achieve this, the algorithm must have at least one input parameter, such as the position where a specific graph transformation should occur or where a particular fragment should be inserted. To make the generation algorithm random, the input parameter(s) must be randomized. However, simply choosing the parameter uniformly from a known set does not guarantee that the output of the generation algorithm satisfies the uniformity condition specified in (1).

### 3.1 Naive Approach

A straightforward approach based on a given generating algorithm is as follows: first, utilizing the given algorithm generate all fullerene isomers with *n* vertices, and enumerate them from 1 to iso(*n*). Then, choose a uniformly random integer between 1 and iso(*n*) to represent a uniformly chosen fullerene isomer.

While theoretically sound, this method is practically limited by the computational difficulty of generating all fullerenes, in particular when *n* > 400. The high computational cost arises from the rapid growth of iso(*n*), coupled with the computational costs of known generating algorithms.

### 3.2 Generating Algorithms for Fullerenes

Many generating algorithms for fullerenes are based on *growth operations* (or *expansions*), and their inverse, *reductions*. Growth algorithms typically begin with a small *seed fullerene*, such as the dodecahedron *C*_20_ or the *C*_24_-isomer, the smallest fullerene with a nonzero number of hexagons. These algorithms incrementally add hexagons, thereby increasing the number of vertices. Notable examples of growth operations include those presented in ref (31), which form the basis of the *buckygen* software;^13^ the operations defined by Buchstaber and Erokhovets,^18^ which generalize the *Endo-Kroto operations* from ref (21) and are further discussed in a broader framework in ref (17,25) the *leapfrog operations* described by Andova et al.^2^ and Coxeter’s operations outlined in ref (37). Importantly, Brinkmann et al.^14^ demonstrated that no finite set of *closed* growth operations can generate all possible fullerene isomers. Here, an operation is called *closed* if its application to a fullerene always results in another fullerene.

It is worth noting that the growth operations proposed by Buchstaber et al.^18^ form a finite family sufficient to construct all fullerenes starting from the dodecahedron *C*_20_. However, these operations are not closed, as they may produce intermediate graphs with one exceptional face, a quadrangle or a heptagon, before ultimately yielding a fullerene. If an algorithm can generate all possible fullerenes, we refer to it as *complete*.

While growth algorithms are efficient for generating fullerenes with a small number of vertices (*n* ≤ 400), their performance declines significantly as *n* increases. This inefficiency is exacerbated by the fact that growth operations are not injective, meaning multiple operation sequences can lead to the same fullerene, resulting in a high rejection rate and increased computational costs.

**Figure 2:**
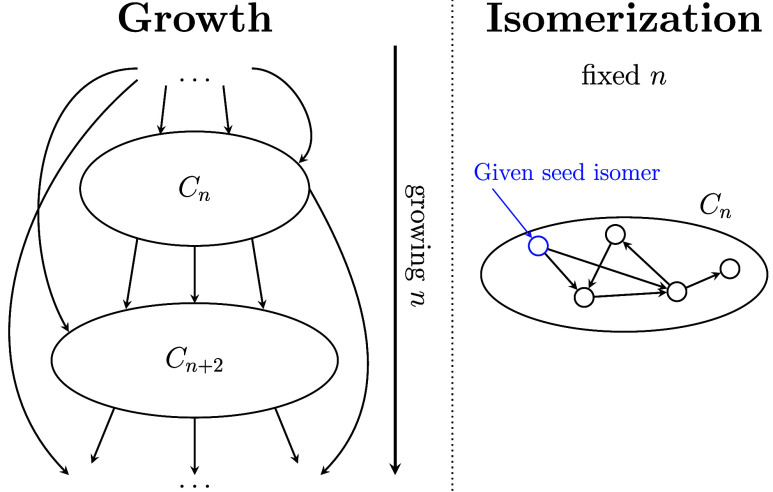
Schematic illustration of growth and isomerization algorithms. Edges represent the connections between two fullerenes via a growth or isomerization operation. The edges are directed (indicated by arrows) since these operations are generally not bijective.

Alternatively, *isomerization algorithms* preserve the number of vertices while rearranging the existing pentagons and hexagons within the fullerene (Figure 2). The most well-known isomerization algorithm is based on the Stone–Wales operations, initially introduced in ref (45). Another example is the set of rotation and mirror operations on the fullerene graph, as introduced by Astakhova and Vinogradov.^3^

While more computationally efficient than growth algorithms, isomerization algorithms rely on the availability of a seed fullerene in *C*_*n*_ beforehand. One approach to generate such seed fullerenes is through growth algorithms. Another way involves dividing the set of all feasible *n* into congruence classes and, for each of these classes, defining a scalable fullerene structure–such as a (*p, q*)-nanotube with a chiral vector depending on the congruence class–that can be expanded to every element of the congruence class based on its vertex count. For example, for all *n* = 20 + 10*r* with , a (5, 0)-nanotube can be constructed similarly to the (5, 5)-nanotubes described in ref (10). In the case of (5, 0)-nanotubes, the cap is composed exclusively of six vertices of degree 5, corresponding to six pentagons in the direct fullerene graph. Consequently, the hexagonal belt contains five vertices, compared to ten vertices in the (5, 5)-nanotubes. Another method for generating seed fullerenes for every *n* ≥ 34 is discussed later in this section.

The *face spiral algorithm*, one of the earliest methods for generating fullerene structures, is neither a growth algorithm nor an isomerization algorithm. Originally proposed by Manolopoulos et al.,^38^ a generalized approach—proven to be complete—was later presented in ref (50) and implemented in ref (43).

### 3.3 Face Spiral Acceptance–Rejection Algorithm

While implementation^43^ represents the most efficient version of the spiral algorithm, it is significantly outperformed by buckygen, currently the most efficient algorithm for fullerene construction. Nevertheless, the spiral algorithm is valuable for its potential to generate a uniform distribution within *C*_*n*_.

The main idea behind the algorithm is to peel a fullerene like an orange, starting from one face and moving through each successive face in a tight spiral manner. As this spiral progresses, one records whether each face is a pentagon or a hexagon, producing a sequence of 5′s and 6’s. By repeating this process in each of the possible 6*n* ways (since a *C*_*n*_-isomer has 3*n* facets as potential starting points of the spiral, and two possible directions for each facet), a list of many sequences is generated. The lexicographically smallest spiral is then selected. The positions of the 12 5′s in this spiral provide the locations of the pentagonal faces yielding a final sequence of length 12 representing the fullerene.

Listing fullerenes in *C*_*n*_ in lexicographical order, based on the positions of their pentagons within the spiral, provides a compact and interpretable representation and enumeration of fullerenes. To randomly select a fullerene from *C*_*n*_ (each isomer having the same probability of being chosen), one can choose a random integer *N* between 1 and  (the total number of all possible positions of pentagons in ascending order), and then retrieve the *N*th sequence from the set of all position vectors.

While generating these sequences can be efficiently realized, their number of length *n* with 12 5′s as coordinates compared to the relatively small number  of fullerene isomers results in a high rejection rate, making this approach impractical. For instance, for *n* = 60, there are  possible pentagon position sequences, but only 1812 isomers in *C*_60_. This results in an acceptance rate of approximately 10^–9^. As the binomial coefficient  grows asymptotically as *n*^12^ due to *Stirling*’*s formula*,^1^ and therefore, faster than iso as *n* → ∞, the acceptance rate diminishes even further for larger *n*.

The first known fullerene to violate this spiral rule is a *C*_380_-isomer, with a second counterexample found among more than 90 billion *C*_384_-isomers. However, all other isomers of *C*_*n*_ with *n* ≤ 450 follow this rule, cf. refs (36,52).

Although the authors of ref (50) proposed clever methods to prefilter sequences which do not correspond to fullerenes, the acceptance rate remains too low to make this approach feasible for large *n*.

Despite these challenges, the spiral method is still widely used for enumerating fullerenes and other polyhedral structures, as it can be extended to all cubic polyhedral graphs, cf. ref (50). Therefore, when referring to *C*_*n*,*j*_, we mean the *j*th isomer in the lexicographical order of all *C*_*n*_-isomers, based on their smallest pentagonal position sequence.

The introductory question of this section remains valid and can be reformulated as a problem statement in the following way:

**Open Problem 1.***Develop a computationally efficient algorithm to generate a uniform distribution on fullerene isomers C*_*n*_*for all feasible n*.

The Stone–Wales operation, an isomerization operation discussed in the following section, has the potential to lead to a solution to this open problem.

## 4 Stone–Wales Operation

In 1986, A. J. Stone and his student D. J. Wales introduced a flip operation on four faces of fullerene graphs, now widely known as the Stone–Wales (SW) operation.^45^ This operation gained popularity among chemists very fast. In their work, Stone and Wales calculated the so-called π-*electron-energy* for various *C*_60_-isomers, generated by applying SW operations to the Buckminster structure *C*_60,1812_.

In chemistry, SW operations are frequently referred to as Stone–Wales defects, and their relevance extends beyond fullerenes to other carbon-based molecular systems.^12^

From a theoretical perspective, the concept of simple yet general flip operations on planar graphs dates back to at least 1936. As proven by Wagner, any two triangulations of the sphere with the same number of vertices and edges can be transformed into one another through a finite sequence of edge flips.^49^ Negami later extended Wagner’s result, generalizing edge flips on triangulations to more complex surfaces.^39^ The operations discussed in this section can be regarded as specific instances and multiple applications of these broader edge flip transformations.

Currently, no closed and complete isomerization algorithm fullerenes is known, although several generalizations of the SW operation have attempted to combine both properties. Two such generalizations–the *polyhedral Stone–Wales* and the *generalized Stone–Wales* (gSW) operation–are introduced in this section. In both cases, the classical SW operation is presented as a special instance of these generalizations.

For the gSW operation, we prove that infinitely many fullerene isomers cannot be generated by any sequence of gSW operations, offering insights into the algorithm’s incompleteness and potential strategies for overcoming it.

### 4.1 Polyhedral Stone–Wales Operation

The pSW operation was introduced by Plestenjak et al.^41^ as part of an effort to generate fullerenes at random.

This operation is also commonly referred to as *Berge*’*s 2-switch operation*, cf. refs (7,40), and it can be regarded as the most general operation within the family of edge-flipping operations.

**Figure 3:**
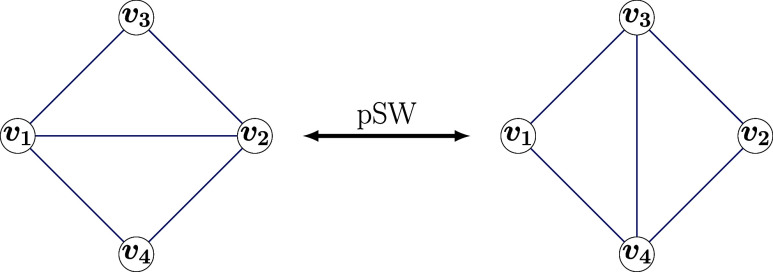
pSW operation on four vertices with arbitrary degrees. If *v*_1_ and *v*_2_ are restricted to be of degree 6, and *v*_3_ and *v*_4_ to be of degree 5 on the left-hand side, the pSW operation is equivalent to the SW operation.

It modifies triangulations by selecting two adjacent vertices, *v*_1_ and *v*_2_, and the edge (*v*_1_, *v*_2_) connecting them in *T*_*n*_. Since *T*_*n*_ is a triangulation, there are exactly two other vertices, *v*_3_ and *v*_4_, that are adjacent to both *v*_1_ and *v*_2_. The pSW operation removes the edge (*v*_1_, *v*_2_) and adds a new edge connecting *v*_3_ and *v*_4_ akin to an edge flip. Figure 3 illustrates this process. While the pSW operation maintains the number of vertices, it does not preserve the degrees of the vertices, meaning the resulting graph may no longer be a fullerene. Therefore, the pSW is not closed with respect to the set of fullerenes but rather operates on the whole set of triangulations with *n*/2 + 2 vertices.

Nevertheless, based on Wagner’s result from ref (49), this set of operations can eventually generate all *C*_*n*_ isomers. Thus, the pSW operation is complete.

The usual SW operation is a special case of the pSW, in which *v*_1_ and *v*_2_ are required to have degree six, while *v*_3_ and *v*_4_ must have degree five. In this case, the two hexagonal vertices become pentagonal, and the two pentagonal vertices become hexagonal after applying the pSW operation.

As noted by Plestenjak et al. in the conclusion of ref (41), it remains unclear how the choice of the seed fullerene affects the distribution of the generated fullerene isomers at the end of the algorithm. In their article, they used the *n*/2-gonal prism, which can be constructed for any feasible *n* and has the same number of vertices, edges and faces as an *C*_*n*_-isomer. The choice of vertices for applying the pSW operation is partly random and based on a specific energy function for polyhedra. Broadly speaking, this selection rule aims to minimize the energy of the polyhedra under consideration. If multiple choices of edges result in the same energy level, an edge is chosen uniformly at random.

### 4.2 Generalized Stone–Wales Operation

In 1995, Babić et al.^4^ introduced another generalization of the Stone–Wales operation, simply termed *generalized Stone–Wales* (gSW) operation, which applies to arbitrary large fragments of fullerenes with a specific structure.

Let the length of a path on a graph be defined as |*V*| – 1, where *V* denotes the set of vertices in the path.

**Definition 1.***For a feasible n and*, *w* ≥ 2, *let a C*_*n*_-*isomer with dual graph T*_*n*_*be given. We call*

(a) *a path* (*v*_1_, ..., *v*_2*w*_) *with v*_*i*_ ∈ *V*(*T*_*n*_) *a gSW path of length* 2*w* – 1, *if the following in T*_*n*_*holds:*
  - *v*_1_*and v*_2*w*_*have degree 5*,
  - *v*_2_*and v*_2*w*–1_*have degree 6*,
  - *v*_*i*_ ≠ *v*_*j*_*for i* ≠ *j*,
  - (*v*_*i*_, *v*_*i*+2_)∈ *E*(*T*_*n*_) *for all i* = 1, ..., 2*w* – 2.
(b) *a subgraph of T*_*n*_*induced by a gSW path, a gSW fragment*.
(c) *a function F*: *C*_*n*_ → *C*_*n*_, *which modifies the edges of T*_*n*_, *such that* (*v*_2_, *v*_1_, *v*_4_, *v*_3_, ..., *v*_2*w*_, *v*_2*w*–1_) *forms a gSW path in F*(*T*_*n*_) *if and only if* (*v*_1_, *v*_2_, ..., *v*_2*w*_) *is a gSW path in T*_*n*_, *a gSW operation*.

**Figure 4:**
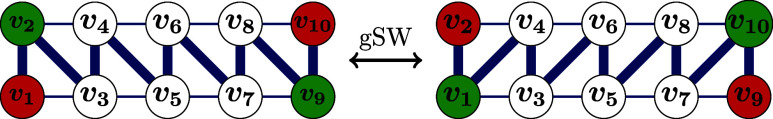
gSW operation on a gSW path with ten vertices (*w* = 5) with thick edges representing the gSW path necessary for the gSW operation.

From this definition, it follows that the existence of a gSW path in a fullerene is necessary to apply the gSW operation to this fullerene. An example of a gSW path and gSW fragment with *w* = 5 is shown in Figure 4. It is also worth noting that the gSW operation is self-inverse. The original SW operation is a special case of the gSW operation with *w* = 2, meaning that no intermediate vertices exist between the first and last pair of vertices. Since the pentagons and hexagons at the beginning and end of the gSW path in a fullerene switch places, and the degrees of all intermediate vertices remain the same, the gSW operation guarantees that the output is again a fullerene. Hence, the operation is closed with respect to the set *C*_*n*_.

On one hand, the gSW operation extends the pSW operation, as both effectively involve edge flipping, though the pSW operation is restricted to flipping a single edge at a time. On the other hand, the gSW operation imposes specific degree constraints on the first and last two vertices in the gSW path. Future studies could investigate relaxing these constraints to develop a hybrid approach that integrates elements of both operations. While such a combined algorithm would no longer be closed within the set *C*_*n*_, it shows potential for significantly reducing computational costs.

The gSW operation is sometimes referred to as the *linear generalized Stone–Wales operation*,^40^ in contrast to the *radial generalized Stone–Wales operation*. The latter represents a further generalization, involving a specific combination of multiple pSW or Berge’s 2-switch operations performed simultaneously. Loosely speaking, the linear generalized Stone–Wales operation extends the SW operation in terms of its length. In contrast, the radial gSW is extended both in length and width, which lies beyond the scope of this discussion.

The authors of ref (4) identified only one isomer with fewer than 70 vertices that lacks a gSW path. Based on this observation, they conjectured that gSW operations might be powerful enough to generate (almost) all fullerene isomers. However, due to this first counterexample, an algorithm solely based on the gSW operation clearly cannot be complete. The number and structure of fullerenes that cannot be generated by a gSW operation have yet to be fully classified, and a proper classification might provide insights into how to overcome this incompleteness.

We demonstrate that infinitely many isomers exist for which gSW operations cannot be applied. By computer experiments, we identified five counterexamples with fewer than 100 vertices, and two with fewer than 70 vertices. The specific counterexamples with *n* ≤ 100 are *C*_20_, *C*_56,622_, *C*_80,31924_, *C*_92,39303_ and *C*_96,191839_. Since the authors of ref (4) briefly mention their counterexample without specifying the number of vertices, it is unclear which specific isomer they refer to. However, they likely exclude the dodecahedron *C*_20_, as it is a trivial counterexample due to the absence of hexagonal faces.

In the following, we construct an infinite family of fullerenes that lack gSW paths, focusing only on paths on the subgraph *T*_*n*_^6^. If we restrict the vertices *v*_3_, ..., *v*_2*w*–2_ to be elements in *V*(*T*_*n*_^6^), a gSW path can be described as a zigzag path of odd length in *T*_*n*_^6^, starting and ending at boundary vertices, i.e., vertices with degree less than six. Hence, we aim to define a structure for the connected components of *T*_*n*_^6^ that permits only zigzag paths of even length.

By studying the aforementioned counterexamples, we deduced a specific structure of the subgraph *T*_*n*_^6^ that does not permit a gSW path and remains consistent under scaling, allowing for the construction of an infinite set of counterexamples. For *n* ≤ 100, based on our numerical investigations, we claim that the five aforementioned counterexamples are the only isomers that do not allow for a gSW operation. For *n* > 100, however, additional counterexamples with different structures may exist, posing an open problem for future research:

**Open Problem 2.***Identify all fullerenes that do not permit a gSW operation.*

To provide a partly solution to this question, we need the following terms.

**Definition 2.***For a feasible n*, *let T*_*n*_^6^*be the hexagonal dual graph of a fullerene in C*_*n*_, *and let*, *r*_1_ ≥ *r*_2_ ≥ *r*_3_, *r*_*i*_ + *r*_*j*_ ≤ *t* – 1 for *i* ≠ *j. We call a connected component of T*_*n*_^6^

(a) *a t-triangle*, *for t* = 0, *if it is a single vertex, and for t* > 0, *if*
  - *it has t*^2^*triangles as inner faces, and*
  - *it has**vertices, three of which are of degree two*, 3(*t* – 1) *vertices of degree four, and the remaining vertices of degree six. The vertices of degree two are called corners of the t*-*triangle*.
(b) *a* (*t*, (*r*_1_, *r*_2_, *r*_3_)) *-triangle*, *if it is a t*-*triangle in which the vertices of r*_1_-, *r*_2_-, *and r*_3_-*triangles at the corners have been deleted. Edges between vertices whose degree decreased due to the deletion are called open edges*.

**Figure 5:**
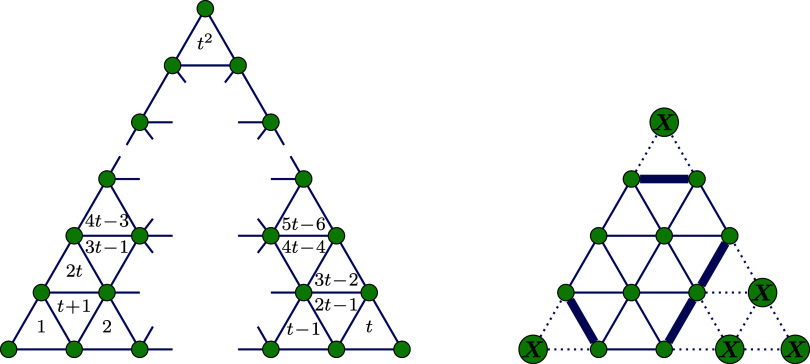
Left: General structure of a *t*-triangle. Right: A (4, (1,0,0))-triangle with deleted edges (dotted lines) and deleted vertices (labeled *X*). Thick lines represent open edges.

The general structure of *t*-triangles and an explicit example of a truncated 4-triangle are illustrated in Figure 5.

As it turns out the hexagonal subgraphs *T*_*n*_^6^ of every counterexample can be partitioned into a set of *t*-triangles and (*t*, (*r*_1_, *r*_2_, *r*_3_))-triangles. For the construction of this partition, we first need to introduce the following terms.

**Definition 3.***A vertex v* ∈ *V*(*T*_*n*_^6^) *is called a 2-facet vertex or 3-facet vertex*, *if v is a boundary vertex of two or three facets of T*_*n*_^6^*larger than a triangle, respectively*.

**Figure 6:**
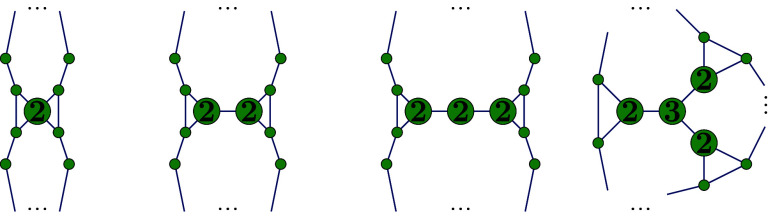
Cases of structures of *T*_*n*_^6^ with 2-facet and 3-facet vertices (labeled 2 and 3, respectively).

From the definition, it follows immediately that a 2-facet vertex has at least degree two and at most degree four, and that a 3-facet vertex must have degree three. Further, since *T*_*n*_ consists of triangles only, a 2-facet vertex of degree four is a vertex of exactly two triangles as illustrated in Figure 6.

The main idea of the following construction is to “cut” the graph *T*_*n*_^6^ along its 2-facet and 3-facet vertices into *t*-triangles and (*t*, (*r*_1_, *r*_2_, *r*_3_))-triangles, such that there are no 2-facet and 3-facet vertices and no vertices with degree 5 anymore.

**Construction 1.***Let T*_*n*_^6^*be the hexagonal subgraph of a dual fullerene T*_*n*_. *Transform T*_*n*_^6^*by applying the following two operations:*

1. *For every 2-facet vertex v* ∈ *V*(*T*_*n*_^6^), *apply the following cut operation:*
  (i) *Choose a minimal set of edges incident to v such that deleting these edges makes v a boundary vertex of only one facet larger than a triangle.*
  (ii) *After deleting these edges, insert a new vertex ṽ into the graph.*
  (iii) *Reinsert the removed edges so that ṽ becomes the end point of these edges, replacing v*.
  *Denote the resulting graph by*.
2. *Let**be all vertices of degree 5 in*. *While vertices of degree 5 exists, perform the following procedure:*
  (i) *Find a pair of vertices* (*v*_*i*_, *v*_*j*_), *i*, *j* = 1, ..., *l*, *i* ≠ *j*, *on a connected component CC of*, *such that the shortest path p* = (*v*_*i*_, ..., *v*_*j*_) *between them is minimal among the shortest paths between any other pairs of vertices of degree 5, and such that the edges of p surround solely triangular facets. The path p divides CC into two parts, denoted by CC*_1_*and CC*_2_.
  (ii) *Delete all edges with one end point in, say, CC*_2_, *and the other end point being a vertex on the path p*.
  (iii) *For every vertex v in p*, *add a new vertex ṽ to*.
  (iv) *Add edges between these new vertices, replicating the edges of the original path p*.
  (v) *Reintroduce all edges deleted in step (ii), replacing the original end point v with its corresponding copy ṽ*.

**Figure 7:**
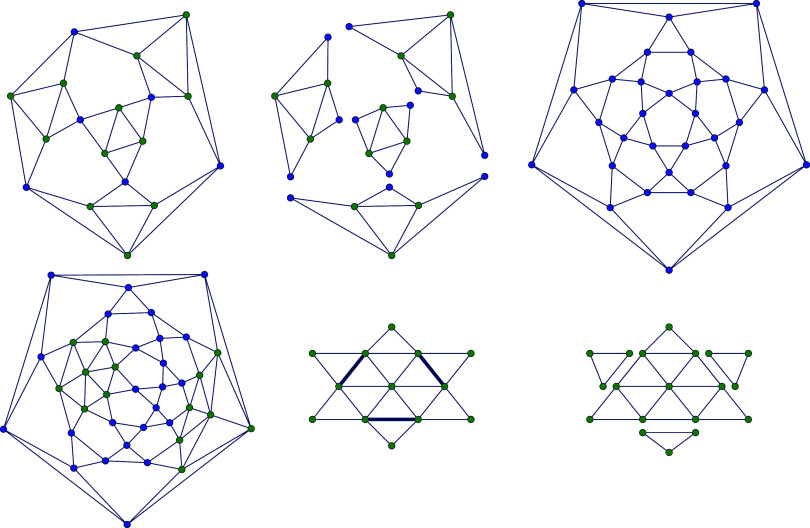
*T*_56,622_^6^ (upper left), *T*_80,31924_^6^ (upper right) and *T*_96,191839_^6^ (lower left) with 2-facet vertices colored blue. The cut-partition of *T*_56,622_ yields four 2-triangles, and *T*_80,31924_ yields 20 1-triangles. Applying the first part of Construction 1 to *T*_96,191839_^6^ produces 12 1-triangles and two copies of the structure in the lower center. Second part of the construction decomposes each of these copies into a 3-triangle and three 1-triangles. Bold edges represent the path ***p***.

Figure 7 illustrates both parts of Construction 1 applied to the hexagonal subgraphs *T*_56,622_, *T*_80,31924_ and *T*_96,191839_^6^.

**Figure 8:**
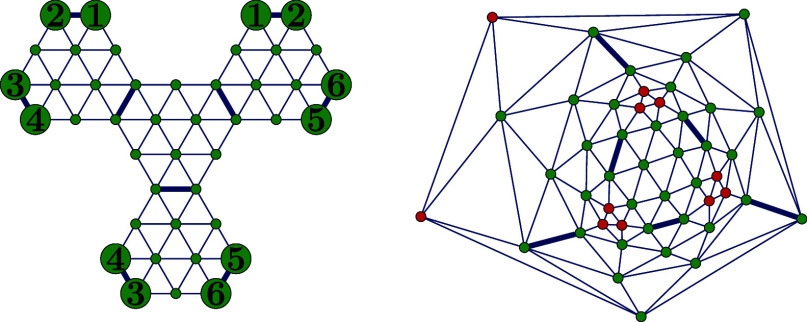
Hexagonal graph *T*_92,39303_^6^ (left) and dual graph *T*_92,39303_ (right), where four (4, (1, 1, 1))-triangles have been glued together along the thick edges. We identify vertices labeled with the same number.

**Definition 4.***For a given T*_*n*_^6^*we call the set of components of the graph generated following Construction 1 a cut-partition of T*_*n*_^6^.

Based on these definitions and our numerical results, we now present the following conjectures.

**Conjecture 1.***C*_56,622_*and C*_80,31924_*are the only fullerenes whose cut-partition consists of t*-*triangles only*.

More importantly, we propose the following

**Conjecture 2.***Let T*_*n*_^6^, *for a feasible n*, *be the hexagonal subgraph of T*_*n.*_*The cut-partition of T*_*n*_^6^*consists solely of elements that are either t*-*triangles or* (*t*, (*r*_1_, *r*_2_, *r*_3_))-*triangles if and only if T*_*n*_*contains no gSW path–with the exception that the cut-partition contains only 0-triangles.*

These two conjectures now lead naturally to

**Open Problem 3.***Prove or falsify Conjectures 1 and 2*.

We now present an algorithm for constructing infinitely many fullerenes that do not contain a gSW path (Figure 8).

**Construction 2.***For an integer t* ≥ 2, *consider four* (2*t*, (*t* – 1, *t* – 1, *t* – 1))-*triangles, glued together along their open edges to form a connected graph, which is the hexagonal subgraph T*_*n*_^6^*of the final dual fullerene. Then, T*_*n*_^6^*has four hexagonal and t*^2^ + 6*t* – 3 *triangular facets, and a total of* 2(*t*^2^ + 6*t* + 2) *vertices. The degrees of the boundary vertices of the four hexagonal facets alternate between 4 and 5. By inserting a 1-triangle consisting of pentagonal vertices into each of these hexagonal facets, the structure ultimately forms a dual fullerene in C*_*n*_*with n* = 4(*t*^2^ + 6*t* + 7).

The key idea behind the proof of the following result is the observation that no dual fullerene constructed according to Construction 2 allows for the existence of a gSW path.

**Theorem 1.***There exist infinitely many fullerenes on which a gSW operation cannot be applied.*

*Proof.* Let , *t* ≥ 2. For *n* ≔ 4(*t*^2^ + 6*t* + 7), let *T*_*n*_ be obtained via Construction 2. Denote by *T*_*n*_^5^ and *T*_*n*_^6^ the pentagonal and hexagonal subgraphs of *T*_*n*_, respectively. Here, we distinguish vertices in *T*_*n*_ by their degree: vertices of degree five are labeled *p*, those with degree six are labeled *h*, and vertices whose degree is unspecified are labeled *v*.

**Figure 9:**
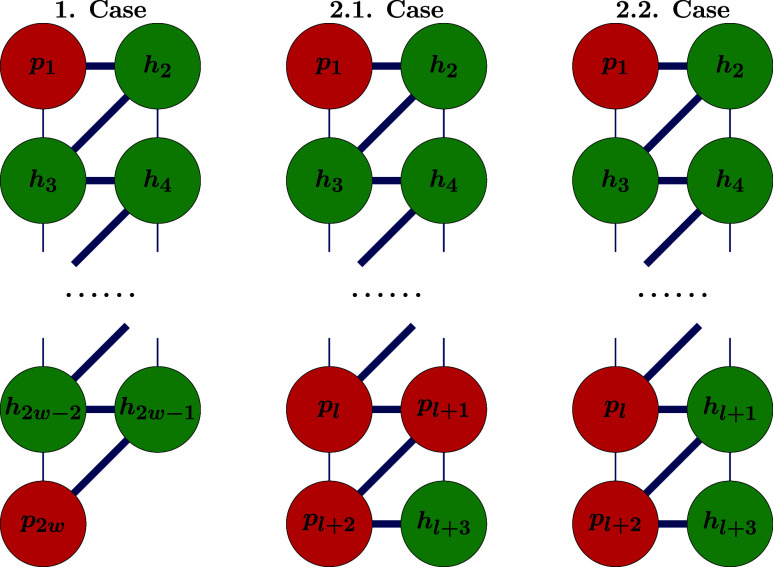
Zigzag path starting with a vertex of degree 5 followed by a vertex of degree 6 in *T*_*n*_ (resulting from Construction 2) can never be a gSW path.

Now assume, for , *w* ≥ 2, that the dual fullerene graph *T*_*n*_ contains a gSW path
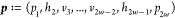
which is a zigzag path of odd length 2*w* – 1, a requirement for a gSW operation. Since *p*_1_, *p*_2*w*_ ∈ *V*(*T*_*n*_^5^) and *h*_2_, *h*_2*w*–1_ ∈ *V*(*T*_*n*_^6^), the two vertices *h*_2_ and *h*_2*w*–1_ must be boundary vertices of two hexagonal facets of *T*_*n*_^6^. We now consider two cases, which are illustrated in Figure 9:

**1. Case:** All the intermediate vertices *v*_3_, ..., *v*_2*w*–2_ of ***p*** are vertices of *T*_*n*_^6^, i.e., *v*_*j*_ ∈ *V*(*T*_*n*_^6^) for every *j* = 3, ..., 2*w* – 2. Then the truncated zigzag path (*h*_2_, ..., *h*_2*w*–1_) lies entirely in *T*_*n*_^6^, and its length 2*w* – 3 is odd. However, by construction, any zigzag path starting and ending at boundary vertices of two distinct hexagonal facets in *T*_*n*_^6^ has even length. This can be verified for *t* = 2 in Figure 8. Hence, the gSW path cannot exist in this case, and no gSW operation can be applied.

**2. Case:** There exists a minimal index *l* ∈ {3, 2*w* – 2} such that *v*_*l*_ ∈ *V*(*T*_*n*_^5^), meaning that vertex *v*_*l*_ has degree 5 in *T*_*n*_. In this case we label it *p*_*l*_ instead of *v*_*l*_.

Since the segment (*h*_2_, *h*_3_, ..., *h*_*l*–1_) lies entirely on *T*_*n*_^6^, it has even length *l* – 3, so *l* must be odd. Given that *T*_*n*_^5^ consists of four connected components, each forming a 1-triangle (triangular facet), we now consider the following two subcases depending on the degree of the next vertex in the path:

**2.1. Case:** If the next vertex *p*_*l*+1_ has also degree 5, then *v*_*l*+2_ must also have degree 5, as the vertices in *T*_*n*_^5^ are arranged in 1-triangles, and *T*_*n*_ has solely triangular facets. The zigzag path (*p*_1_, *h*_2_, ..., *p*_*l*_, *p*_*l*+1_, *p*_*l*+2_) is then of even length *l* + 1. The remaining part of the path (*p*_*l*+2_, *h*_*l*+3_, ...,, *h*_2*w*–1_, *p*_2*w*_) is also of even length 2*w* – *l* + 5, contradicting the requirement that a gSW path must have odd length. Hence, ***p*** cannot be a gSW path. If additional intermediate vertices *p*_*l*_1__, ..., *p*_*l*_*u*__ of ***p*** belong to *V*(*T*_*n*_^5^), the same reasoning can be applied inductively, since the segment (*p*_*l*_*j*_+2_, *h*_*l*_*j*_+3_, ..., *p*_*l*_*j*+1__) has the same structure as (*p*_1_, *h*_2_, ..., *p*_*l*_1__).

**2.2. Case:** If the next vertex *h*_*l*+1_ ∈ *V*(*T*_*n*_^6^), then *v*_*l*+2_ must belong to *T*_*n*_^5^, and due to the structure of *T*_*n*_^5^, the next vertex must have degree 6. The segment of ***p*** starting from *p*_*l*+2_ has the same structure as (*p*_1_, *h*_2_, ..., *p*_*l*_1__) from the previous subcase, leading to the same contradiction.

In all cases, we deduce that no gSW path can exist in *T*_*n*_. Therefore, the gSW operation cannot be applied, proving the theorem.

According to the definition of the gSW operation in ref (40). Theorem 1 has been proven only for *linear* gSW operations. However, a numerical experiment suggests that the *radial* gSW operation is also not applicable to *C*_92,39303_, the first example in the family of fullerenes used in the proof of Theorem 1 and generated by Construction 2. This implies that the set of gSW operations must be extended by at least one additional generalization to achieve completeness. Extending Theorem 1 to radial gSW operations and identifying a new generalization of the SW operation applicable to fullerenes generated by Construction 2 will be a crucial step toward solving Open Problem 3.

**Figure 10:**
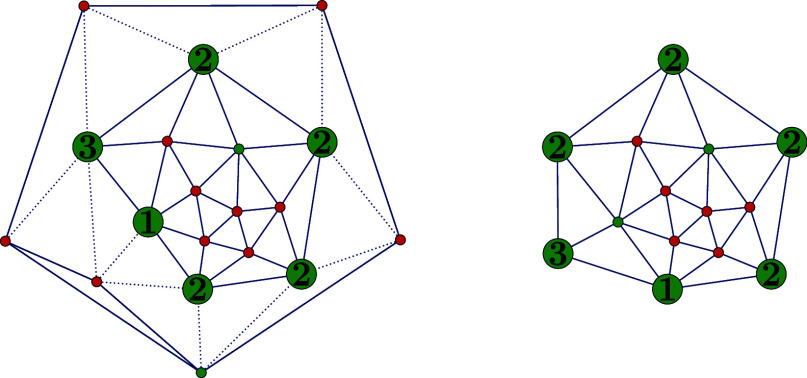
Left: Dual fullerene graph *T*_36,1_. Isomer *C*_36,1_ can serve as an initial structure for generating seed fullerenes of arbitrary size for any isomerization algorithm. Right: The inner connected component obtained by applying Construction 3 to *C*_36,1_ once with the unchanged boundary degree sequence preserved. Replacing the corresponding component in *C*_36,1_ with this component yields a dual fullerene graph in *C*_38_.

Finally, let us discuss a simple construction of seed fullerenes in *C*_*n*_ with *n* ≥ 36 which may be used as a starting point of some isomerization algorithm. Upon analyzing our data, we observed that for every 36 ≤ *n* ≤ 150, there exists an isomer whose pentagonal arrangement resembles the structure shown in Figure 10. Indeed, it can be proved inductively that such a fullerene exists for any *n* ≥ 36. The construction outlined below forms the main idea for proving this, and it also suggests how an efficient algorithm for generating a seed fullerene for a given *n* ≥ 36 might be structured.

**Construction 3.***Starting with C*_36,1_, *remove all dotted edges shown on the left in*Figure 10. *Label each boundary vertex v of the larger connected component (the one with more vertices) with* 6 – *d*(*v*), *where d*(*v*) *is the degree of vertex v. This results in the lexicographically smallest label vector (1, 2, 2, 2, 2, 3) for the six consecutive boundary vertices. Next, by adding a new vertex and connecting it to the vertex labeled 1 and its adjacent vertices labeled 2 and 3, a connected component is obtained with an additional vertex while preserving the same label vector as shown on the right in*Figure 10. *Afterward, the dotted edges can be reintroduced, resulting in a C*_38_*fullerene*.

*This procedure can be iterated, as the label vector and its length remain unchanged throughout. This implies that the modified, larger connected component can be reconnected with the other, smaller connected component. By repeating the process n*/2–18 *times, a C*_*n*_-*isomer for any feasible n ≥* 36 *can be efficiently constructed*.

## 5 Spectra of Fullerenes

Next, we aim to describe the shape of a fullerene using the spectra of its adjacency and degree matrices. The spectral analysis of graphs based on various matrix representations has a long-standing tradition, as seen in refs (5,15,16).

### 5.1 Characters as Unique Fullerene Descriptors

In ref (11),^11^ the authors uncovered a notable connection between the (chemical) relative energy of fullerene molecules *C*_60_ and *Newton polynomials of order*, which are defined as
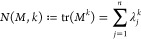
where λ_*j*_, *j* = 1, ..., *n*, are the eigenvalues of a matrix *M* representing a dual fullerene graph *T*_*n*_.

Common choices for *M* include the adjacency matrix *A*, the degree matrix *D*, or a linear combination of those α*A* + β*D* with . Alternatively, instead of the full dual fullerene graph *T*_*n*_, one may focus on subgraphs such as *T*_*n*_^6^ or *T*_*n*_^5^, and their associated matrices. For instance, the study in (11) focuses on the adjacency matrix of the hexagonal subgraph *T*_*n*_^6^, where the authors observed a Pearson correlation coefficient between Newton polynomials *N*(*M, k*) and the relative energy of the molecules often exceeding 0.9, depending on the order *k*. This high correlation, along with the energy-based ordering of fullerenes outlined in ref (46), allows for the identification of energetically stable isomers at a reduced computational cost. The authors chose hexagonal subgraphs partly because they conjectured that the positions and arrangements of the remaining 12 pentagons could be inferred from larger-than-triangle facets in *T*_*n*_^6^ and the degree sequences of their boundary vertices.

Notably, when M = *A*, the Newton polynomials *N*(*A, k*) represent the number of closed paths of length *k* on the graph with adjacency matrix *A*. This interpretation reveals that Newton polynomials of varying orders *k* encode specific structural information about the fullerene, such as the number of edges when *k* = 2. This suggests the potential to combine Newton polynomials across all orders into a comprehensive graph descriptor that uniquely characterizes the fullerene.

**Definition 5.***For a feasible n*, *let T*_*n*_*be a dual fullerene graph, and A and D its adjacency and degree matrices. For*, *we call*
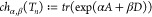
*the* (α, β)-*character of T*_*n*_, *where the matrix exponential is defined as*
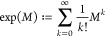
*for every complex-valued quadratic matrix M*.

Note that the (α, β)-character can be rewritten as
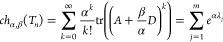
where λ_1_, ..., λ_*m*_ with *m* = *n*/2 + 2, are the eigenvalues of *A* + β/α*D*. This representation reveals that the character is essentially an infinite series of Newton polynomials of the adjacency matrix *A* + β/α*D*. Analyzing the eigenvalues of *A* + β/α*D* becomes sufficient to compute the (α, β)-character.

The behavior of the (α, β)-character varies considerably with different choices of parameters α and β, raising the important question of identifying an appropriate range of parameters. Extensive numerical experiments for various combinations of α, β have led us to the following statement.

**Conjecture 3.***For* α, β ∈ (0, 1) *and feasible n*_1_, *n*_2_, *let T*_*n*_1__, *T*_*n*_2__*be two dual fullerenes. It holds that*
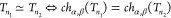


Therefore, the following is presented for future research:

**Open Problem 4.***Prove or falsify Conjecture 3*.

As the number of vertices in a fullerene tends to infinity, the fullerene structure becomes composed solely of hexagonal facets. Two notable configurations of infinitely many hexagons are the *hexagonal lattice* (also referred to as *graphene*) and *infinite (p, q)-nanotubes*. For precise definitions and further details on their eigenvalues, we refer to ref (9) for the hexagonal lattice and ref (10) for the infinite (*p*, *q*)-nanotubes. In general, the dual of a 3-regular infinite graph *H*, composed only of hexagonal facets, is an infinite triangulation *T*. If a loop of weight 3 is added to every vertex of *T*, then the average number of closed paths of length *k* per vertex, denoted by μ_*k*_(·), satisfies the following relation:
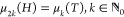
2

This observation connects to a similar result for finite fullerene graphs. As conjectured in ref (9),^9^ a sequence of dual fullerene graphs, represented by the matrix *A*_*n*_ + 1/2*D*_*n*_ can be constructed such that their normalized Newton polynomials of degree *k* converge to μ_*k*_(*T*) as *n* → ∞:
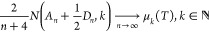
For their duals, i.e., the direct fullerene graphs represented by *A*_*n*_, it follows that
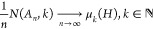


These observations, along with Conjecture 3, suggest the parameter choice α = 2β = 1/2 when analyzing dual fullerene graphs, and α = 1, β = 0 for direct fullerene graphs. Henceforth, whenever the parameters (α, β) are omitted, we refer to the (1/2, 1/4)-character. In addition to the unique characterization of fullerenes in Conjecture 3, the characters seem to induce an ordering within the set of all fullerenes that is reflexive, transitive, antisymmetric, and total.

### 5.2 Linear Ordering of Fullerenes and Their Limiting Shape

Provided that Conjecture 3 holds true, an enumeration method based on characters is injective, which is a necessary condition for linearity. A second desirable property for enumeration is the monotonicity with respect to the number of vertices, meaning that
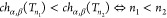
3

When both α and β tend to 0, the (α, β)-character converges to the number of vertices in the corresponding dual graph:
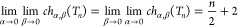
Thus, condition (3) is satisfied for sufficiently small α, β. However, if α and β are too small, the numerical precision required to distinguish between different fullerenes in *C*_*n*_ increases, leading to higher computational costs, as the characters must be computed with greater accuracy.

On the other hand, the character grows exponentially with α and β, which can result in the smallest character in *C*_*n*_ becoming smaller than the largest character in *C*_*n*–2_. In other words, for a feasible *n*, the range of characters *ch*_α_,β in *C*_*n*_ expands with increasing α and β. Therefore, the parameters must be chosen small enough to prevent overlapping in the character ranges for different *n*, ensuring that condition (3) is maintained.

These considerations highlight the importance of selecting appropriate values for α and β to balance computational efficiency while ensuring that condition (3) is fulfilled.

**Figure 11:**
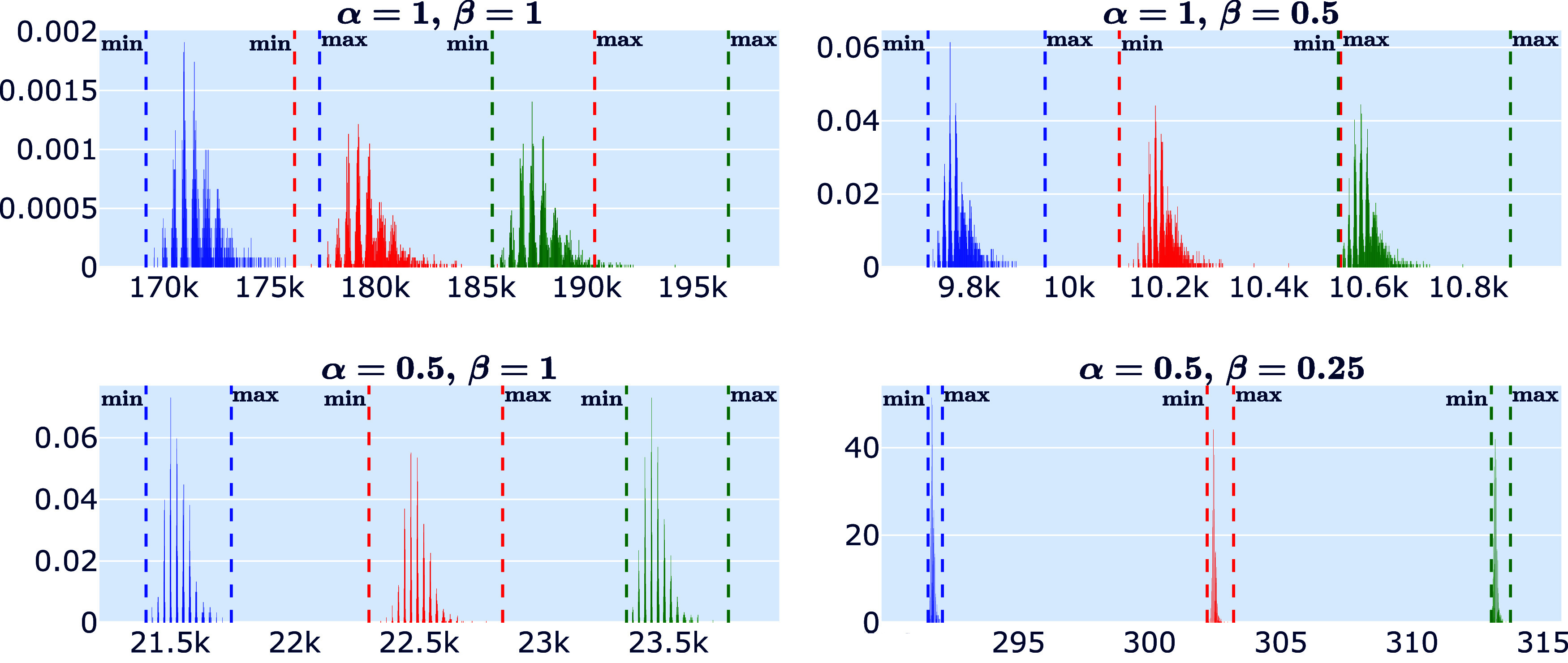
Ranges of (α, β)-characters of *C*_58_ (blue), *C*_60_ (red), *C*_62_ (green) with different parameters (α, β).

In Figure 11, histograms of the (α, β)-characters are shown for four combinations of (α, β) and *n* ∈ {58, 60, 62}. The vertical dotted lines mark the minimal and maximal character for each *C*_*n*_, with blue, red, and green lines representing *C*_58_, *C*_60_, and *C*_62_, respectively. In the upper-left plot with α = β = 1, the character ranges overlap, with the largest character in *C*_58_ not only surpassing all characters in *C*_58_ by far but also the smallest character of *C*_60_. This overlap is significantly reduced for (α, β) = (1, 1/2), as seen in the upper-right plot, though a small intersection remains between *C*_60_ and *C*_62_, caused by the appearance of the (5, 0)-nanotube in *C*_60_, which has an disproportionately large character. For (α, β) = (1/2, 1), the ranges no longer overlap, but the character values grow significantly, even for small *n*, as indicated by the ticks on the *x*-axis. Thus, computing characters and enumerating fullerenes with these parameter values would demand significant computational power as *n* increases. The lower-right plot shows more distinct character ranges for the chosen values α = 2β = 1/2 with a more practical character scale, supporting the choice of parameters in this study.

Additionally, extreme fullerene structures were observed to have the largest and smallest characters. In particular, (*p*, *q*)-nanotubes typically exhibit higher characters, where smaller circumferences *p* + *q* correspond to larger characters. This relationship arises from the fact that nanotubes with smaller circumferences permit a greater number of closed paths of a given length resulting in a higher character. For example, the (5, 0)-nanotube provides an upper bound for the character within *C*_*n*_. As detailed in ref (10) and as discussed in Section 3.2, a (5, 0)-nanotube exists for every *n* = 20 + 10*r* with .

In contrast, *Goldberg polyhedra*, i.e., fullerenes with icosahedral symmetry (highest symmetry a fullerene can attain), tend to have the smallest characters. A Goldberg polyhedron exists in *C*_*n*_ if two integers  satisfy *n* = 20((*p* + *q*)^2^ – *pq*), cf. ref (26), making the Goldberg polyhedron a nanotube with chiral vector (5*p*, 5*q*), cf. ref (10), corresponding to the largest feasible circumference in *C*_*n*_.

In cases where a feasible *n* allows integers  to satisfy the equation *n* = 20 + 10*r* = 20((*p* + *q*)^2^ – *pq*), meaning that *C*_*n*_ contains both a Goldberg polyhedron and a (5, 0)-nanotube (structures with extreme characters), these two can be utilized to centralize and normalize the characters of all dual fullerenes within *C*_*n*_.

A fundamental question that arises here is how these centralized and normalized characters are distributed over the interval [0, 1].

**Open Problem 5.***Check the convergence of*
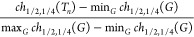
4*as n* → ∞, *where G represents a dual fullerene graph in C*_*n*_, *and T*_*n*_*a random dual fullerene graph in C*_*n*_. *If the limit exists, find its explicit form*.

**Figure 12:**
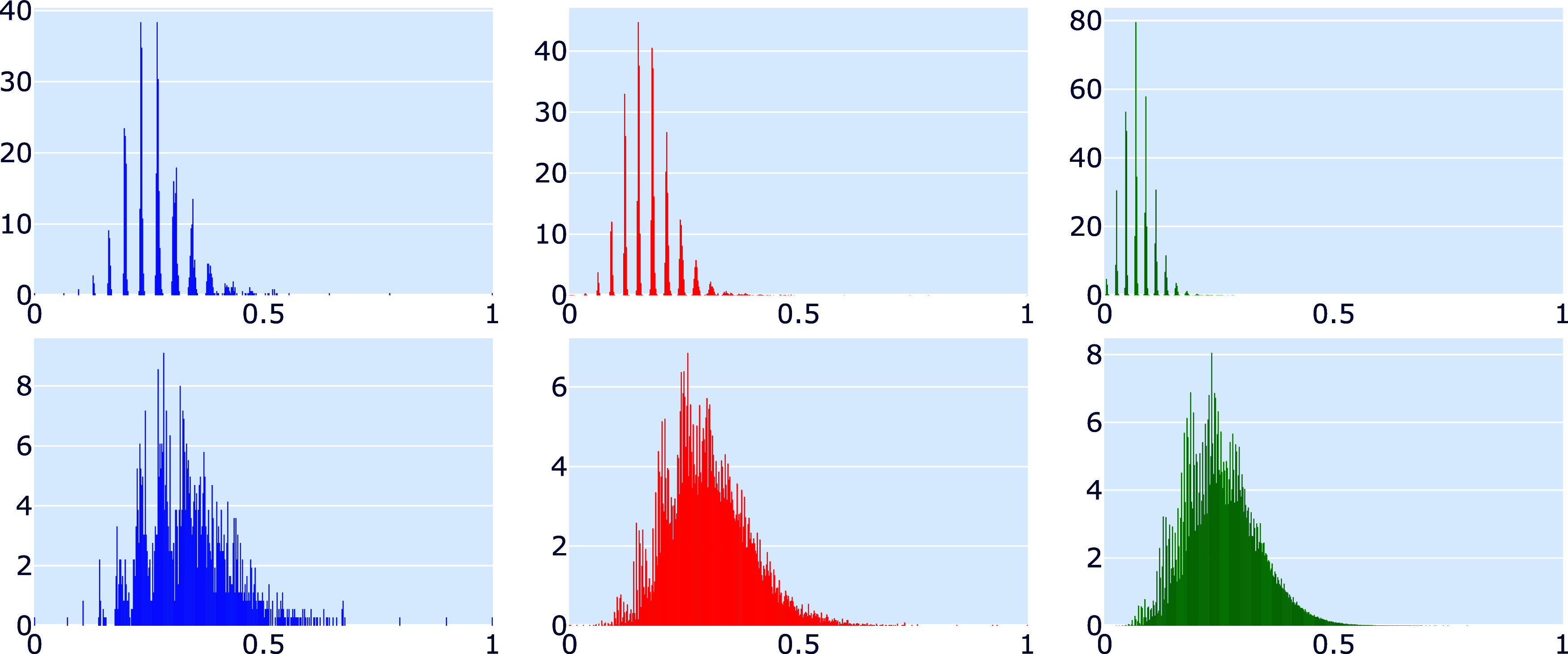
Normalized histograms (1000 bins) of characters of isomers *C*_*n*_, centered and normalized as per (4), for *n* = 60 (blue), *n* = 80 (red) and *n* = 140 (green) using *T*_*n*_ (upper row) and *T*_*n*_^6^ (lower row).

One possible starting point for addressing this problem is Figure 12, which shows the empirical densities of characters adjusted according to (4) of all *C*_*n*_-isomers for *n* = 60, 80, 140. It appears that as *n* grows, the normalized histograms tend to converge to a Γ-shaped limiting distribution density.

Understanding the asymptotic behavior of expression 4 could provide insights into generating large random fullerene isomers by sampling from the limiting distribution of their centralized and normalized characters.

Since dual fullerenes consist solely of vertices of degree six in the limit *n* → ∞, it may be advantageous to analyze the same distribution using the hexagonal subgraph *T*_*n*_^6^ instead of *T*_*n*_.

## 6 Summary

This paper explores several open mathematical problems related to fullerenes, with a primary focus on their generation, enumeration, and spectral properties. It articulates several key problem statements and conjectures, summarized at the end, which call for further investigation.

The paper begins by tackling the challenge of generating random fullerene isomers, offering an overview of current generating algorithms. While the face spiral conjecture implies an established method for enumerating fullerenes, it fails to yield a uniform distribution over *C*_*n*_, leaving open the problem of how to achieve such uniformity.

Next, the discussion shifts to a generating algorithm based on the gSW operation, revealing an infinite family of fullerenes for which this operation is proven to be inapplicable. Numerical examples illustrate the structural limitations of the gSW operation, particularly for fullerenes with fewer than 100 vertices.

Additionally, the introduction of a novel graph invariant for fullerenes, termed the *character*, derived from adjacency and degree matrices, marks another significant contribution. This invariant conjectures a linear ordering of all fullerenes, offering an alternative framework to the face spiral method for enumeration and structural analysis of fullerenes. Conjecture 3 and the investigation of the limiting behavior of the centralized and normalized characters, as given in expression 4, pose fundamental questions regarding the uniqueness of the character and its applicability to fullerene ordering and generation.

To summarize, this paper outlines the following five open problems that might motivate future research:

1. Develop a computationally efficient algorithm to generate a uniform distribution on fullerene isomers *C*_*n*_ for all feasible *n*.
2. Identify all fullerenes that do not permit a gSW operation.
3. Prove or falsify Conjectures 1 and 2 regarding the relationship between cut-partitions and the gSW operation.
4. Prove or falsify Conjecture 3 about the uniqueness of (α, β)–characters.
5. Determine, if existent, the distribution of centered and normalized characters (4) of random fullerenes as *n* → ∞.

## Data Availability

The numerical results presented in this article can be reproduced using Python notebooks available at ref (8).
